# 3D Visual Reconstruction as Prior Information for First Responder Localization and Visualization

**DOI:** 10.3390/s23187785

**Published:** 2023-09-10

**Authors:** Susanna Kaiser, Magdalena Linkiewicz, Henry Meißner, Dirk Baumbach

**Affiliations:** 1 German Aerospace Center (DLR), Institute of Communications and Navigation, Oberpfaffenhofen, D-82234 Wessling, Germany; 2 German Aerospace Center (DLR), Institute of Optical Sensor Systems, D-12489 Berlin, Germany; magdalena.linkiewicz@dlr.de (M.L.); henry.meissner@dlr.de (H.M.); dirk.baumbach@dlr.de (D.B.)

**Keywords:** indoor navigation, pedestrian dead reckoning, simultaneous localization and mapping, FootSLAM, collaborative mapping, professional use cases, prior maps, 3D building reconstruction

## Abstract

In professional use cases like police or fire brigade missions, coordinated and systematic force management is crucial for achieving operational success during intervention by the emergency personnel. A real-time situation picture enhances the coordination of the team. This situation picture includes not only an overview of the environment but also the positions, i.e., localization, of the emergency forces. The overview of the environment can be obtained either from known situation pictures like floorplans or by scanning the environment with the aid of visual sensors. The self-localization problem can be solved outdoors using the Global Navigation Satellite System (GNSS), but it is not fully solved indoors, where the GNSS signal might not be received or might be degraded. In this paper, we propose a novel combination of an inertial localization technique based on simultaneous localization and mapping (SLAM) with 3D building scans, which are used as prior information, for geo-referencing the positions, obtaining a situation picture, and finally visualizing the results with an appropriate visualization tool. We developed a new method for converting point clouds into a hexagonal prism map specifically designed for our SLAM algorithm. With this combination, we could keep the equipment for first responders as lightweight as required. We showed that the positioning led to an average accuracy of less than 1m indoors, and the final visualization including the building layout obtained by the 3D building reconstruction will be advantageous for coordinating first responder operations.

## 1. Introduction

In the field of indoor pedestrian navigation, the use of building information is advantageous, especially for locating pedestrians and visualizing their locations. Highly accurate positioning is mandatory for professional use cases, which are the focus of this paper, without excluding other applications. For instance, in a police operation like a hostage situation, a rampage, or a terror attack, the commander wants to know the locations of the team members to coordinate their actions. Another example is a building on fire, where knowledge about the locations and activities of the first responders (FRs) supports the coordinator in reacting quickly if, for instance, an FR or an injured person inside the building needs help. For this, a situation picture including the geographical location and the nature of the building and the outdoor environment is of interest for the commander of the FRs. During an operation, the planning of the actions as well as the localization of the FRs can be enhanced by knowing the building in a geo-referenced way. In this work, the main objective was to provide an overall situation picture, including a 3D reconstruction, accurate FR localization, and visualization, bearing in mind the FR requirements when designing the system to be worn by each FR. The 3D reconstruction is foreseen to be performed in advance or, in the future, on site within minutes, and localization can be performed in real time. The localization system can additionally exploit the information from the 3D reconstruction—if available—for enhancing positioning accuracy. The main focus of this paper was to obtain a 3D reconstruction of a whole building, measure its quality, apply this information in an appropriate format to a localization technique, and finally analyze the influence of incorrectly reconstructed walls/parts of the building on the positioning accuracy. In the following, we first describe the state of the art for 3D reconstruction methods and localization techniques, followed by analyzing the research gaps for each system.

- **Three-dimensional reconstruction:** If a floorplan or any other kind of information about the building layout is not available, the prior information of the building can be obtained by 3D building reconstruction using different kinds of sensors. For obtaining the outer envelope of the building and the outer environment, a visual or laser sensor can be mounted on a flying vehicle such as a helicopter, an airplane, or even a drone; the building can be scanned by flying over the building; and the visual data can further be processed to provide the outer envelope and environment. Several approaches can be found in the literature for outer reconstruction. In principle, one can distinguish between three different methods [1]: photogrammetry reconstruction, sparse and dense reconstruction based on structure for motion (SfM), and procedural modeling. Cameras, stereo cameras, and laser-based scanners (LiDAR) are used as sensors for 3D reconstruction. A comprehensive overview of the state of the art in outdoor 3D reconstruction can be found in [2], where a multi-drone reconstruction method was also presented. More generally, in [3], a systematic literature review was presented for crime scene reconstruction, where traditional methods like photos, videos, and paper documentation as well as upcoming methods like augmented and virtual reality devices were discussed, and a quality assessment for the different papers was provided.
  Indoor 3D reconstruction methods mainly use visual or laser-based scanners that are either handheld, carried on a helmet, or mounted on a robot. An overview of different methods was provided in [4] for camera-based systems and more generally in [5,6] for different kinds of sensors. Most of the systems have been tested only indoors and/or within only one floor level. For instance, in [7], a system was described where a robot was equipped with a LiDAR device and a camera. Different indoor rooms were scanned and, in parallel, people are detected to account for crowded environments. While detecting people in parallel is surely an aspect to be considered and investigated, the authors only provided results for single rooms. In addition, placing the system on a robotic platform increased the density and decreased the noise of the 3D point cloud calculated from such input data [8]. This could be important for the accuracy and completeness of vector models calculated from such 3D point clouds. Another example is the method described in [9], where a low-cost mobile laser scanner was used for estimating the indoor layout. In this work, available architectural data were used for constraining point clouds. For this, a series of architectural skeleton feature patterns was designed, and a fast localization initialization method was proposed.
  Commercial systems for 3D reconstruction, which can be a combined solution together with localization techniques, rely on a multi-sensor approach where optical sensors are fused with inertial sensors [10], among other sensors. These systems are usually carried by hand or are located on the operator’s body (e.g., helmet systems [11]). Due to their design as end-consumer products and the related black-box concept, the integration of additional information from other data sources (e.g., further sensors or measuring systems) can lead to difficulties.
  3D reconstruction is a process of capturing the shape and appearance of real objects, aiming to create a mathematical model applied in computer vision, virtual reality, robotic navigation, reverse engineering, etc. There are two main approaches to 3D reconstruction: traditional geometric reconstruction and a relatively new deep learning method.
  The classic tool chain for 3D reconstruction from imagery usually starts with image co-registration, followed by bundle block adjustment and subsequent dense matching [12,13], delivering a 3D point cloud. Following this, the derived cloud representation is traversed in voxel space, applying a principle component analysis (PCA) to identify planar structures [14]. Finally, the entire voxel space is analyzed again to connect adjacent and similar parts of the 3D representation. This approach is applicable for both indoor and outdoor reconstruction [15].
  Tool chains to reconstruct 3D scenes using neural networks often have two stages. The first neural network generates the 3D point cloud, e.g., by using the truncated signed distance function (TSDF) [16,17]. Then, a second network is used to identify and extract relevant, representative 3D object content [18,19]. However, there are approaches combining both aforementioned steps in one single neural network [20].
  A comparison between traditional approaches and neural-network-based methods shows that the classical state of the art approach delivers models with superior accuracy, while deep learning tends to improve the completeness of the models [21].
  All 3D reconstruction methods mentioned above consider either the exterior or the interior of the building or environment. An overall situation picture obtained within a short time and estimating the whole building, including all rooms and levels, as well as the outdoor environment so that both fit together in a geo-referenced way has not yet been provided, to the best of our knowledge.
- **Localization:** Besides 3D reconstruction, we also address accurate FR localization in indoor environments in this work. Due to the lack of GNSS signals indoors, various sensors are investigated in the literature, and a sensor fusion approach is usually applied for pedestrian navigation to provide reliable positioning. The different sensors that are used for indoor navigation and can be found in the literature and on the market range from inertial and magnetic sensors to using radio frequency signals like WiFi, BLE beacons, RFID, ZigBee, UWB transmitters, and finally to the use of other sensor types like visual sensors, sound sensors, and optical sensors (light, infrared). An overview of different indoor navigation solutions can be found in [22,23], and for smartphones in [24]. Different visual localization techniques are summarized in [25]. Commercial systems include indoo.rs [26], Aionav [27], beQ [28], and Oriient [29]. All systems use either indoor map information, and/or multiple sensors and/or make use of signals of opportunity.
  The following techniques especially designed for FRs in the field of 3D reconstruction and localization can be found in the literature: The authors of [30] provided an overview of applications combining 3D building models and indoor navigation during an emergency. Different indoor localization techniques, including artificial intelligence (AI)-based methods, were presented, and various 3D representations of a building including the Industry Foundation Classes (IFC), a standard for the data exchange of building information modeling (BIM), and 3D visualization techniques were examined.
  In [31], the challenges facing accurate and reliable indoor localization for FRs and soldiers were described, and sensor fusion solutions were analyzed, including inertial sensors and ranging. Pointer [32] is a system whereby 3D positions can be estimated via a low-frequency magnetic field. FRs can be detected within 70 m with an accuracy of below 2 m. In [33], several transceivers and ranging systems were investigated for FRs, and the best transceiver was an ultra-wideband transceiver with a symmetric two-way ranging protocol. According to the authors, this system still needs improvements. A system based solely on inertial measurement units (IMUs) and magnetometer data is the so called PERSY system [34]. However, besides GNSS, PERSY uses a tactical-grade IMU, STIM300, from Sensonor, which is of high cost and therefore may not be affordable if used for a high number of emergency forces. In addition, it cannot be easily enriched with prior map data to enhance the position accuracy.
  The localization systems described above are not fully adapted to the requirements and needs of FRs. They either need infrastructure to be installed, do not work fully in 3D environments, are not applicable for dark and dusty environments, are of high cost, or are heavy and of high computational complexity—which also implies a high rate of battery consumption. Moreover, the influence of erroneous information about the building layout when assisting FR localization and displaying the locations within the 3D environment has not been fully investigated. Finally, in many systems, the data that are transferred to the command center are not restricted, so that communication systems with a high degree of building penetration and subsequent lower data rate cannot be taken into account.

In the application considered here, the localization devices should be adapted to the special needs of the FRs. Because FRs already wear a lot of heavy equipment, it is better to use a system based on small and lightweight sensors that are preferably of low cost. The system should restrict the amount of data that are transmitted to the head of the operation located either at the emergency car or at a central station. It should operate in real time and work as a stand-alone system in case there is no prior map (PM) available in the database, and it should be integrated in clothes, e.g., in a shoe, in a pocket, or on the arm. Furthermore, it should work with fast FR speeds, and all movements of the FR should be captured. The visual sensors used for 3D reconstruction could also be considered for localization, but they present the following disadvantages: (i) in zero-vision situations like fog and smoke, they do not provide reasonable results, (ii) the expense of data processing and transmission has to be considered, (iii) the weight and calibration of the sensors are significant, and (iv) data protection has to be kept in mind. For radio frequency solutions, specific infrastructure like a network of passive elements or transmitters needs to be installed—i.e., they are not infrastructure-less. In an emergency case, infrastructure cannot be installed, and it cannot be assumed that it already exists, especially in the emergency scenarios that we considered: for instance, in tunnels, forts built in the mountains, mines, coves, or even buildings on fire. Therefore, we excluded radio frequency solutions. Of all other sensor types, the most suitable sensor for emergency applications is the inertial sensor, as it does not rely on, e.g., the lighting conditions, which would make visual and optical sensors inappropriate. The systems for real-time 3D reconstruction and localization based on visual/optical sensors are often computationally complex, requiring a fast CPU with a lot of memory that also presents a high rate of battery consumption. Furthermore, it is usually not practical to send a huge amount of data (like with visual sensors) from inside buildings through many walls or even stone to the central station. Privacy issues must be considered when using body cameras, and occlusion and changing objects within the environment makes visual/optical localization even harder. In the following, we describe the systems applied in this paper for 3D reconstruction and localization. We point out why we selected the respective systems.

- **Three-dimensional reconstruction:** For outdoor reconstruction, we used a fully automated system to generate scaled maps on demand and on-site within minutes. This system is named a Modular Aerial Camera System (MACS) [35]. It can, for instance, be installed on a vertical take-off and landing (VTOL) drone. It uses a traditional approach with high accuracy. Knowledge about the indoor situation is usually provided by scanning the building with a camera system or a laser scanner. In this paper, a multi-sensor approach including a low-cost inertial measurement unit combined with a stereo camera system and an optional tilt sensor was used for this purpose. This system is named an Integrated Positioning System (IPS) [15]. Both the outer envelope and environment provided by the MACS and the inner layout provided by the IPS were combined and used as prior information for indoor FR localization. The 3D building reconstruction could be scanned and pre-computed in advance and is foreseen to be stored in a database for the emergency services. Our approach would create a complementary database for the 3D reconstruction of the inner and outer shells of a building by fusing data from a handheld and a flying system [36]. The reason for choosing this automated system for 3D reconstruction was that it has already been tested in different real-world scenarios like earthquakes for estimating a situation picture. It has a high TR level and accuracy. It is not a black-box system like commercial systems, i.e., it could be adapted to our needs. Finally, it works in 3D environments with different floor levels and combines the inner layout with the outer building envelope, providing a situation picture of the whole area.
- **Localization:** The real-time indoor navigation system proposed in this paper is the so called NavShoe in combination with FootSLAM. The NavShoe is based on a single inertial sensor unit. The 3D gyroscope and 3D accelerometer sensor data are used as input for a 15-state unscented Kalman filter (UKF). The NavShoe outputs the drifted position and heading of the FR or pedestrian. More information about the NavShoe can be found in [37]. The FootSLAM algorithm is cascaded to the NavShoe to reduce the remaining drift and to learn a map of walkable areas inside the building. FootSLAM is based on a Rao-Blackwellized particle filter and learns walkable areas by counting the transitions of edges in a hexagonal prism map, weighting with transitions counters, and exploiting the fact that transitions in floors and rooms are usually repeated. FootSLAM can be enriched using PMs and fusing additional sensors like altimeters, GNSS receivers, magnetometers, or signals of opportunity (WiFi/UWB); it can consider known locations or learn places/features by marking them; and it can be used collaboratively (FeetSLAM) either after receiving whole walks/walk parts or successively. A comprehensive description of the principle of FootSLAM can be found in [38] and the citations therein. Neither system, NavShoe or FootSLAM, relies on any available infrastructure. The NavShoe can be realized with an inertial sensor connected to a mini CPU and is foreseen to be integrated into a shoe in future, for instance in the sole of a shoe or at the ankle of an FR. The FootSLAM algorithm itself can be realized within the central station using a more powerful CPU. Within this system, the amount of data to be transmitted to the central station is restricted to time, position, and some necessary flags like a stairs indicator estimated by the NavShoe, and these data only need to be sent every second. We chose this system because it is of a low cost and light weight; has already been tested with FRs; has a high TR level; is robust against sensor drifts; is extendable for exploiting other sensor signals; and was designed for a low rate of data transmission, which will play an important role in the choice of communication systems in the future.

The novelty of this paper lies in the combination of prior maps resulting from a separate, accurate 3D building reconstruction with a SLAM and a visualization framework for coordinating FRs. This combination makes the overall system very attractive, because one can enhance FR localization, only limited data need to be transmitted to the central station, and the computational cost of the device worn by the FR is kept very low. Second, the method for converting the 3D reconstruction information into a suitable PM in 3D for the SLAM is new. This process involves the creation of a 3D layout from the point cloud, including door detection; the extraction of a semantic floor plan from this layout; the conversion of the floor plan with the diffusion algorithm into a probabilistic presentation based on angular PDFs; and, finally, the conversion of the angular PDFs into a hexagonal prism map that is suitable for the SLAM. The 3D layout extraction is subsequently helpful in the final 3D visualization tool. Third, our investigation of the performance using different prior information—i.e., a 3D reconstructed map, a known map, and no initial map but the learning of a map of walkable areas during a walk with the SLAM algorithm—in combination with the overall system approach including FR tracking and 3D visualization within a 3D reconstructed geo-referenced multi-floor environment is novel.

The paper is organized as follows: The overall system with its components of 3D outdoor building reconstruction, 3D indoor building reconstruction, and the conversion of building information into a FootSLAM PM is described in Section 2. Section 3 shows the building reconstruction results and the resulting PMs and provides localization error rates for the experimental walks depending on the PMs used as input to FootSLAM. A discussion of the results is provided in Section 4. Finally, Section 5 is devoted to conclusions.

## 2. System Description

### 2.1. Overview of the System

**Figure 1:**
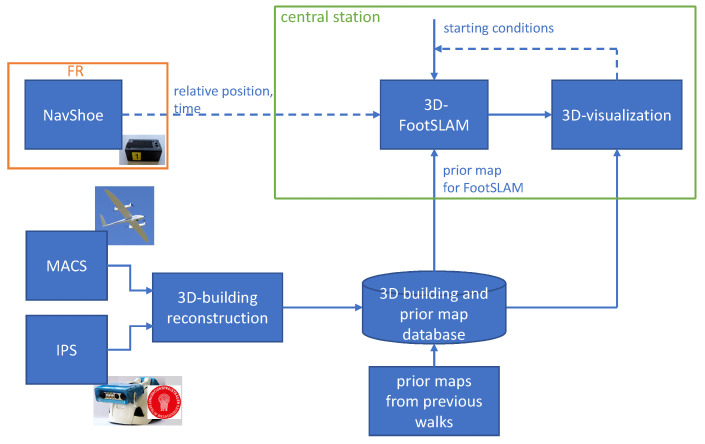
Overview of the whole system, with the FR-related NavShoe system indicated within the orange box and the FootSLAM and visualization systems at the central station—e.g., an emergency car—shown within the green box. The 3D building reconstruction can be performed in advance or on-site. The 3D building and prior map database can be located either at the central station or in an external database like a cloud.

The overall system is depicted in Figure 1 and will be explained in the following. First, the building envelope is reconstructed with the MACS. For this, the building envelope is scanned with an unmanned aerial vehicle (UAV) carrying a camera system. The geo-spatial information of the position of the building is estimated simultaneously. The inner building layout is scanned with the IPS. The results from the MACS and IPS are combined to provide a 3D reconstruction of the whole building. This 3D reconstruction is calculated in advance, and the 3D building information can be stored in a database. In the future, the time for the building reconstruction is foreseen to be reduced to approximately 20 min after the measurement walk so that it can also be applied in emergency cases when this information is unknown. After that, the PM suitable for FootSLAM is generated from this information. It can be enhanced with the use of prior information from the FootSLAM system itself. This can be achieved by wearing the NavShoe on the foot and walking through the building with it. The FootSLAM result is a map of walkable areas. The PM resulting from 3D reconstruction and optionally combined with the map of walkable areas is then used within the localization system. It should be noted that all *prior* information is gathered in advance. If the prior information is not available, localization with the NavShoe and FootSLAM will nevertheless be possible, because FootSLAM learns the environment during the walk. At the beginning of the walk, without a PM the positioning will be more unsure because there are no loop closures or revisited areas to correct the drift. In addition, the map can be slightly rotated without the geo-reference of the PM or the use of any GNSS signal—which is foreseen to be fused in the future. If available, the PM resulting from the 3D reconstruction and/or the previous walks of FootSLAM is used as input to FootSLAM for tracking FRs wearing the NavShoe. Finally, the 3D visualizer shows the positions of the FRs within the environment. The NavShoe is foreseen to be worn by each special force, and it will send their positions to the operation center, where FootSLAM is performed and the final positions are visualized. The transmission of the positions to the operation center will be addressed in future research and is therefore not a part of this paper.

### 2.2. 3D Outdoor Building Reconstruction with MACS

The processing tool chain obtaining digital surface models (DSMs) or, in this case, a 3D model of the building from remote sensing data commences by refining the parameters of the exterior sensor orientation (EO) and/or interior camera orientation (IO). Usually, exterior orientation parameters are given with three degrees of freedom (DOF) for translation (*X*,*Y*,*Z*) and three DOF for rotation (ω,ϕ,κ). Common interior camera parameters are the focal length (*f*); principal point ($x_{0}$,$y_{0}$); and several distortion parameters (e.g., the radial symmetric distortion polynomial with ($k_{0}$, $k_{1}$, $k_{2}$, *…*)).

The orientation refinement approach (often called bundle block adjustment, BBA) consists of two steps. Finding unique image features, serving as measurements in the image space, is the first step, and minimizing the distance between backward-projected 3D estimates and previously determined feature positions for homologous features is the second step [39]. The combination of all forward-projected rays, projected from the image coordinate system to the real-world coordinate system for one unique feature, forms the shape of a bundle. Bundle block adjustment is the process of solving a non-linear least-squares problem to deliver a bundle, tightened as much as mathematically possible, whilst refining the IO and EO parameters.

Having obtained all features (in the image space) and the back-projected image coordinates of the 3D estimates, the final step of BBA is minimizing “the reprojection error between the image locations of observed and predicted image points, which is expressed as the sum of squares of a large number of non-linear, real-valued functions” [40]. This process is achieved by a non-linear least-squares algorithm, with the Levenberg–Marquardt algorithm turning out to be the most successful for solving this problem [40]. The final minimization method is described more deeply in [41]. The minimization results are refined model parameters in form of optimized EO and/or IO parameters.

In photogrammetry and computer vision, extracted and refined image features and their projection to the real-world coordinate frame are often referred to as a coarse 3D reconstruction of the observed scene. However, in the context of this paper, it was necessary to deliver a complete reconstruction containing information as fine and accurate as possible. Therefore, another field of research played an important role in the 3D reconstruction workflow, namely dense image matching and subsequent point cloud fusion.

Dense image matching aims at finding a corresponding pixel in a matched image for every pixel in a selected base image. The distance between both pixel locations is called the disparity [42]. Obvious limitations (non-reconstructable areas) are occlusions or areas of both images that do not overlap. There are several techniques to produce a dense disparity representation (map) from two images. A quite simple approach is to use a neighborhood, surrounding the pixel of interest of the base frame, and find the best-matching pixel (out of all pixels) in the match frame. However, this (local) method is prone to blunders, especially for images of highly redundant content or repetitive textures. Extending the local approach to a global one delivers more reliable results but increases the computation significantly [43].

To account for both problems (runtime and accuracy), Hirschmüller [44] proposed “the Semi-Global Matching (SGM) technique, which offers a good trade off between accuracy and runtime and is therefore well suited for many practical applications”.

Starting with rectified images, according to the stereo normal case, for every base frame pixel the matched image is traversed alongside the corresponding epipolar line. At every epipolar line position, the image is scanned at a predefined number of paths (e.g., 8, 16, …). This step expands the technique to a semi global approach. Furthermore, costs are accumulated for every path, with costs defined according to pixel similarity (e.g., the Hamming distance [45] or mutual information criterion [44]). The final step consists of an energy minimization *E* for all accumulated costs $C(p,D_{p})$, where *p* is the current path and $D_{p}$ is the corresponding disparity [44]:

(1) $$E\left(D\right)=\sum_{p}\left(C\left(p,D_{p}\right)+\sum_{q\in N_{p}}P_{1}T\left[|D_{p}-D_{q}|=1\right]+\sum_{q\in N_{p}}P_{2}T\left[|D_{p}-D_{q}|>1\right]\right)$$

Hirschmüller proposed in this energy minimization method two smoothness constraints [44]: the constant penalty $P_{1}$ within the second term was introduced for pixels in the neighborhood, for which the disparity changes a little bit (i.e., by 1 pixel), and the larger constant penalty $P_{2}$ was introduced for large disparity changes. $P_{1}$ permits an adaptation to slanted or curved surfaces, while $P_{2}$ preserves discontinuities. *T* is the probability distribution of corresponding intensities in this equation.

The result of the SGM step is a 3D pointcloud of the observed scene (e.g., a building), which is the basis for subsequent 3D visualization (see Section 3.4).

### 2.3. 3D Indoor Building Reconstruction with IPS

**Figure 2:**
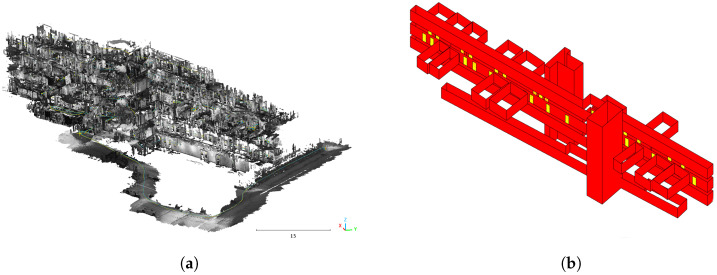
3D building model: Combined IPS 3D point cloud over several floors and an outdoor section (**a**) and the corresponding calculated indoor vector model (**b**).

**Figure 3:**
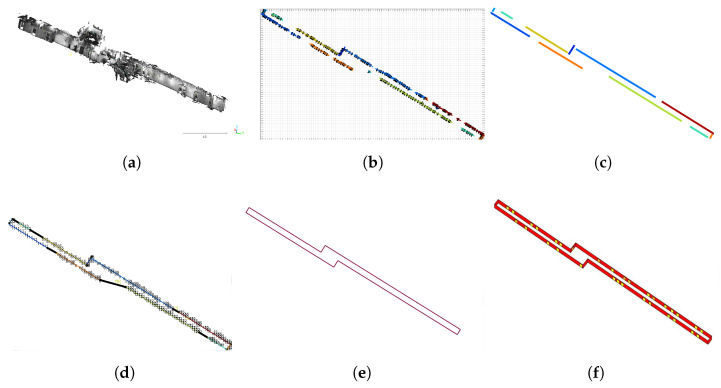
Three-dimensional vector model reconstruction: (**a**) point-cloud voxel projected onto XY plane; (**b**) 2D grid and regression lines in grid cells; (**c**) calculated segments; (**d**) adjacent endpoints of the segments; (**e**) closed contour; (**f**) extruded contour.

In order to calculate a 3D indoor vector model of the building (see Figure 2b), the oriented IPS images are preprocessed, i.e., performing the semi-global matching algorithm [44] to convert them into a 3D point cloud, as shown in Figure 2a. After preprocessing, the resulting high-resolution samples are manually subdivided into floors, rooms, and staircases, as shown as an example in Figure 3a. Next, each 3D cloud is projected onto the x,y−plane subdivided by a regular grid (see Figure 3b). The spacing of the grid needs to be adjusted empirically with respect to the residual noise of the points. For each grid cell its density, spatial distribution, and characteristic height histogram is computed and evaluated to identify potential facade pieces [13]. On the set of cell elements, which is considered a part of the facade, the regression line is estimated from the 2 × 2 covariance matrix of the confined point set. After effectively performing PCA, the eigenvector of the covariance matrix, which belongs to the greatest eigenvalue, denotes the direction of the best-fit line through the set of cell elements. A measure for its confidence can be obtained from the ratio $\rho_{min}=\frac{\sigma_{min}}{\sigma_{max}}$ of the square roots of the eigenvalues, which is the ratio of the standard deviations of the projected 3D points in the direction of the eigenvectors. After the line segments have been determined for individual grid cells, the computation of the regression line is extended to groups of adjacent cells in order to form facade fragments of the same orientation (see Figure 3c). For this purpose, the line direction of every cell in a local neighborhood of eight is compared pair-wise against the line direction of its center element using a fixed angular threshold. In case the directions match, the respective cells are added to the same cell set. This growth process is repeated until no more adjacent cells are left to be assigned. If a stable state is reached, a common regression line is estimated through the point set of every cell group. The resulting linear facade fragments are intersected if their endpoints are locally adjacent (Figure 3d) within the grid, forming a closed two-dimensional contour (Figure 3e). Finally, contour extruding is calculated, as shown in Figure 3f. The complete 3D vector model is shown in Figure 2b.

To reconstruct the doors (yellow lines in Figure 3f), one point of each door is manually measured on the IPS images. Knowing the parameters of the IPS camera and the transformation matrix between the IPS and the model coordinate systems, at first a ray passing through the measured point and the projection center is built. Then, the intersection point between the ray and the 3D vector model is calculated. The intersection point places the door on the 3D model. Each door has the same width and height.

### 2.4. Converting 3D Building Reconstruction Information into a FootSLAM Prior Map

**Figure 4:**
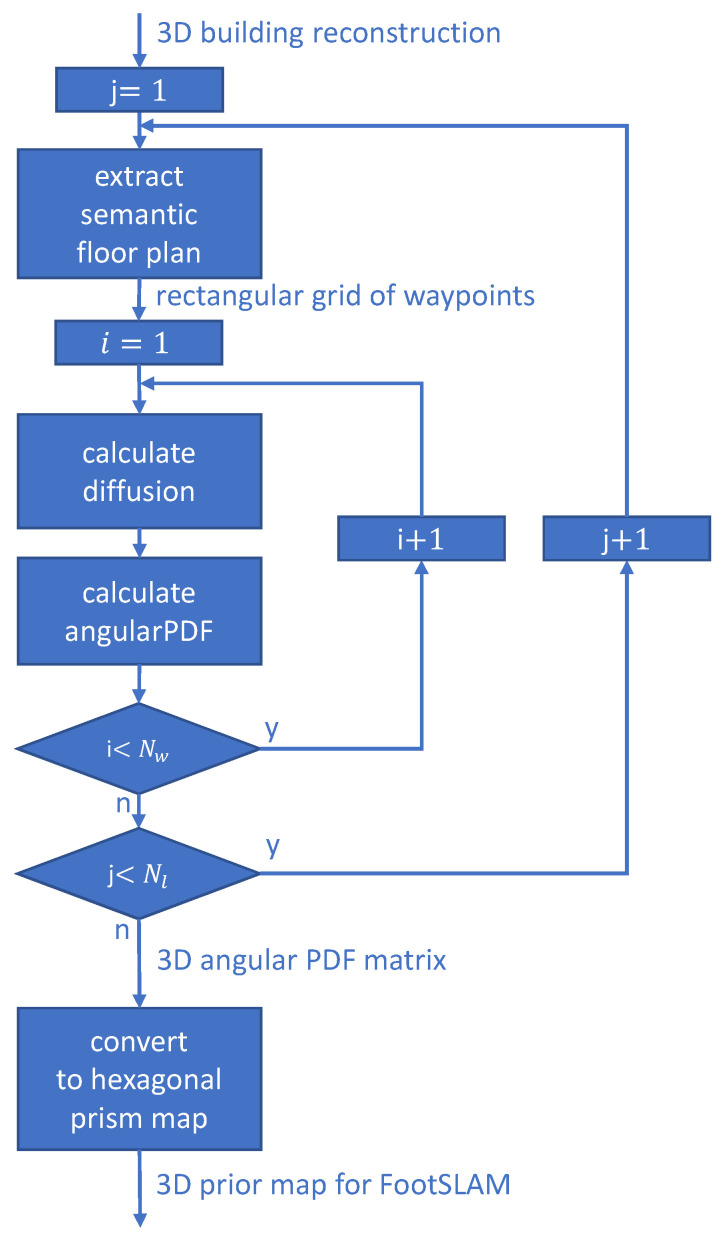
Overview of generating the prior map for FootSLAM. The conversion of the 3D reconstruction data consisted of three main parts: semantic floor plan extraction, 3D angular PDF matrix calculation, and conversion to a hexagonal prism map.

**Figure 5:**
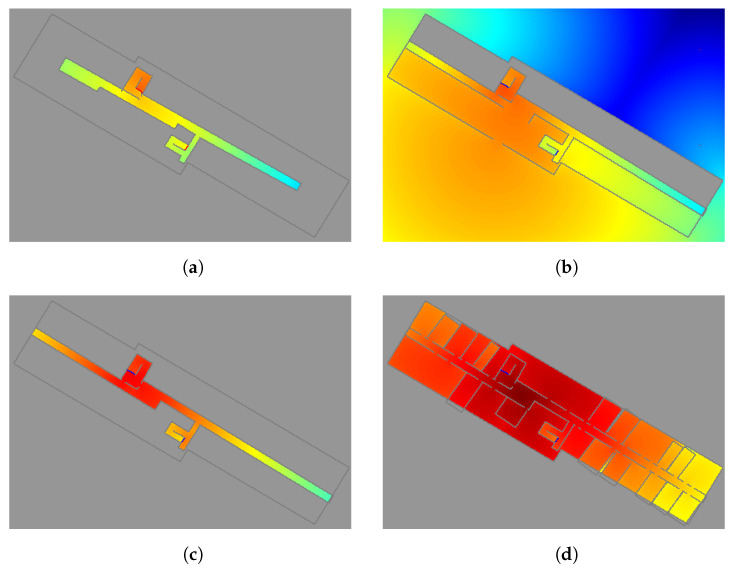
Diffusion results for the basement (**a**), ground floor (**b**), first floor (**c**), and second floor (**d**) of our office building. The diffusion was calculated for a point (given in dark red) in the second floor, and the gas was distributed over different floor levels via the staircase areas. The colors code the gas level values, from high in dark red to low in blue. If closed, the entrances of the stairs (going up) are marked in red and the exits (arriving up) in blue.

**Figure 6:**
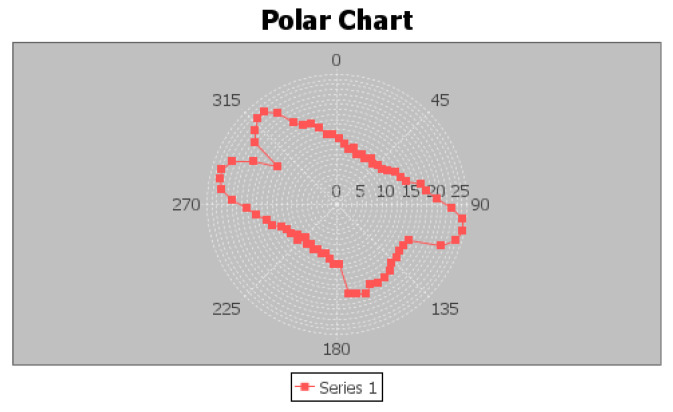
Angular PDF calculated from the gas distribution for the waypoint of Figure 5. The angular PDF was extracted from the gas distribution using the distance to one of the contour lines of the diffusion results. Walkable directions are preferred by applying higher values to the respective direction.

To obtain the PM information for FootSLAM from the 3D building reconstruction (Section 2.2 and Section 2.3) and acquire a geo-referenced map, the extracted building information of the MACS and IPS is used to generate a 2.5D map in a format that is appropriate for FootSLAM. An overview of the process for generating this PM is given in Figure 4. From the information about the walls and staircases extracted from the 3D building reconstruction as a semantic 2.5D floorplan, an angular probability density function (PDF) 2.5D map of the building is first generated. The angular PDFs are calculated by applying the 2.5D diffusion algorithm in a first sub-step [46]. For this, the area of one level $l_{j},j=1\ldots N_{l}$, where $N_{l}$ is the number of floor levels, is divided into a rectangular grid of waypoints represented for simplicity as a vector of waypoints $w_{i},i=1\ldots N_{w}$, where $N_{w}$ is the number of waypoints. For each waypoint $w_{i}$, an angular PDF is calculated to obtain a matrix of angular PDFs for the whole area, where each matrix element corresponds to a specific waypoint $w_{i}$. Figure 5 shows the diffusion results for four floor levels (basement, ground floor, first floor, and second floor) of our office building in Oberpfaffenhofen, where one waypoint in the second floor (dark red point in Figure 5d) is used as a source for calculating the diffusion values—like a source effusing gas. The gas is distributed over different floor levels via the stairs areas. In Figure 5, the colors code the gas level values, from high in dark red to low in blue. It should be noted that we used the floorplan estimated from the 3D reconstruction, where only parts of the building were scanned. In a second sub-step, the angular PDFs are extracted from the gas distribution using the distance to one of the contour lines of the diffusion results [46]. Figure 6 shows the angular PDF results for this specific waypoint (given in dark red in Figure 5d). The angular PDFs are calculated in a 2.5D manner: the gas is further distributed to the other floor levels using the information about the staircase area. The method for further distributing the gas to the other floors is presented in [47]. For this, we closed either the entrance or the exit of the stairs (in Figure 5, the entrances of the stairs (going up) are marked in red and the exits (arriving up) in blue if closed). The whole process is repeated for each floor level $l_{j}$.

In a second step, the 3D angular PDF matrix is converted to a FootSLAM map built on a hexagonal prism map. The 2D method for converting this map is given in [48]. This method was additionally extended in this paper for handling 2.5D maps. The difficulty of extending this method lay within the staircase area. Within the stairs area, the counters of the top and bottom edges of the hexagon needed to be set to suitable values in order to allow for vertical transitions. For this, we divided the stairs area into two halves and calculated the distance from the center of the hexagon to the entrance/exit of the stairs. Depending on the distance, we estimated the height of the prism to be filled with counter values. We filled the six horizontal faces of this hexagonal prism of the estimated height with the respective values of the angular PDF, as in [48]. Additionally, to account for vertical transitions, we set the top and bottom face counters to reasonable values, so that vertical directions were also favored in that area. It should be noted that in our experiments the number of hexagonal prisms between two floors was six.

### 2.5. 3D Visualization

To visualize all results in one viewer, an appropriate visualization framework was identified. The Potree-viewer, developed at Technische Universität Wien, provides all necessary features. This viewer was specifically designed to be “capable of streaming and rendering point cloud data sets with billions of points, without the need to transfer the whole data set first or to install a third-party viewer” [49]. In addition to point clouds, it is also capable of visualizing triangulated meshes. Another feature is that both 3D representations can be opaque, transparent, or something in between, suitable for inspections in a clear spatial context.

The implementation in javascript also enables the real-time visualization of changing content, i.e., moving scene objects or assets. This feature will be particularly valuable in future projects when personnel (e.g., FRs) have to be displayed in real time. Another very important feature of this viewer is that it uses WebGL [50] in order “to put hardware-accelerated 3D content in the browser” and simultaneously provides a degree of platform independence.

## 3. Experimental Results and Discussion

### 3.1. Resulting Prior Maps from IPS and MACS

#### 3.1.1. Technical Specification and Experimental Settings

To obtain the outer envelope of the building, we used MACS-SaR (MACS Search and Rescue) mounted on a VTOL drone and flew over the building area in a measurement campaign. MACS-SaR incorporates an industrial camera, a dual-frequency GNSS receiver including inertial-aided attitude processing (INS), and an embedded computer [51,52]. The camera head consisted of a 16 MPix CCD sensor (ON Semi-conductor KAI-16070 with a Bayer pattern) and an industrial F-Mount lens (Schneider Kreuznach Xenon-Emerald 2.2/50). The aperture was set to f4.0, and the focus was fixed to the hyperfocal distance. The exterior orientation calculation was based on a dual-antenna GNSS receiver (Novatel OEM7720) in combination with an industrial-grade micro electro mechanical systems-inertial measurement unit (MEMS-IMU) (Epson G320N). The dual-antenna setup was used to determine the true heading independently from the INS. This improved the orientation accuracy, in particular when the movement direction and heading did not correlate due to cross-wind. Additionally, the dual-antenna system allowed for very fast attitude initialization on the ground without aircraft movement. The distance (basis) between both GNSS antennas (mounted in the front and tail) was 0.95 m. The GNSS receiver continuously estimated the position and attitude. The end-of-exposure signal was sent to the GNSS receiver.

Thus, every image was assigned a precisely measured time, position, and orientation. Considering the interior camera orientation (long-term stable), direct geo-referencing could be applied. Due to the continuous synchronization of all subsystems, each image could be time-stamped with a precision better than 1 μs. Time synchronization, image acquisition, and real-time image processing were carried out by the embedded computing unit. This computer was powered by a Quad Core Processor (Intel Atom E3950) with 8 GB RAM and ran a Linux operating system. In this configuration, the system allowed us to capture up to four raw images per second, which could be stored on an removable storage device. The weight was 1.4 kg (including the embedded PC, camera, IMU, GNSS receiver, GNSS antenna, power management, and structure), and the dimensions were 10 × 14 × 20 cm^3^.

A fixed-wing drone was used as a carrier, providing a flight time of approximately 90 min at cruise speeds between 60 km/h and 90 km/h [53]. Thus, the carrier was capable of traveling a distance of up to 105 km per battery charge. The maximum take-off weight (MTOW) was specified to be 14 kg, including a payload of up to 2 kg, and it had a wingspan of 3.5 m. It could operate at wind speeds of up to 8 m/s and temperatures between 0 °C and 35 °C. While its typical flight operation altitude was in the range of 100 m to 300 m above ground level, it was capable of operating at altitudes up to 3000 m above sea level.

The operational range was only limited by the maximum flight time because the autopilot system allowed fully automated flights beyond the visual line of sight (BVLOS). This required a predefined flight plan with terrain following mode for security reasons. The drone was equipped with a conventional command and control link as well as an additional mobile network radio for BVLOS operation. For safety reasons, this carrier was equipped with position lights and an integrated automatic dependent surveillance broadcast (ADS-B) transceiver.

The IPS system comprised a sensor head with two industrial panchromatic CCD cameras of type Prosilica GC1380H, an inertial measurement unit (IMU) ADIS-16488, and an inclination sensor ADIS-16209. The resulting point cloud consisted of 54.2 million points. The overall processing time was 2–3 days, because of the combination of MACS and IPS results, floor plan extraction, and door detection. The stand-alone MACS was fully automated. This currently manual process is intended to be reduced to hours or even minutes in the future. The building was scanned with only a few pedestrians walking through the building. If we discovered pedestrians, we held the camera towards walls or the ground.

#### 3.1.2. Evaluation of 3D Reconstruction

**Figure 7:**
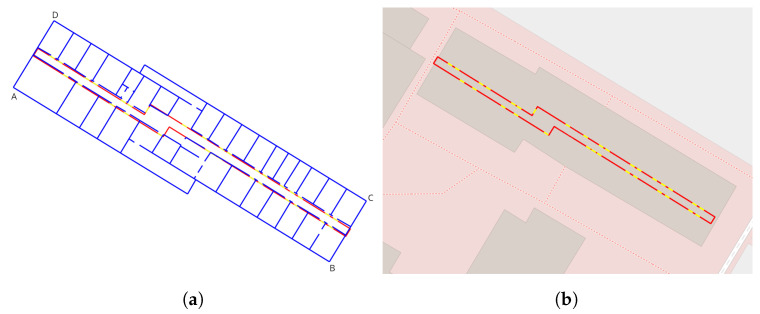
PM evaluation: (**a**) IPS vector model (red) with doors (yellow) overlaid with a 2D reference floor plan (blue); (**b**) absolute accuracy of the 3D vector model (reference: OSM data, ArcGIS Pro (Esri)): red lines—floor, yellow lines—doors.

In a first evaluation step, the relative accuracy of the PM was examined. As shown in Figure 7a, the 3D vector model (red lines) and the doors (yellow lines) generated by the IPS were overlaid with a floor plan (blue lines). The floor plan was generated before the measurement campaign and was used here as a reference. Both corridor models fit on top of each other well. Following the contours of both 2D vector models from left to right, a small rotational error could be seen. In addition, some walls in the middle of the floor were generated incorrectly (compare also Figure 3c,d). These were made of glass, and in combination with difficult lighting conditions this led to incorrect projections and inaccuracies in the 3D reconstruction. Also, 1 out of 32 doors was incorrectly reconstructed. All other doors fit well with the reference data.

In the second evaluation step, the absolute position of the 3D model was analyzed. For this purpose, the model was compared with OSM data in ArcGIS Pro by Esri. Both the model and the OSM data were available in the UTM coordinate system. As Figure 7b shows, the model matched the OSM reference quite well. It should be noted that we don’t know the precision of the underlying openstreet map data.

To obtain a measure of the quality of the reconstruction, we measured the Euclidean distance at the four corners (A, B, C, and D) of the reconstructed building compared to the known building layout (four corners in Figure 7a). The results are given in Table 1. Here, $d_{A}$ is the distance of the bottom left, $d_{B}$ of the bottom right, $d_{C}$ of the top right, and $d_{D}$ of the top left corners. The results showed that the positions of the building corners were very close to the real values of the building. The highest distance was 0.27 m. It should be noted that the real values were the positions in the middle of the walls, and the walls were about 20 cm thick. Moreover, we rotated the building layout with an affine transform, and this may have caused additional uncertainties in the resulting corner positions.

### 3.2. Resulting Combined Maps

**Figure 8:**
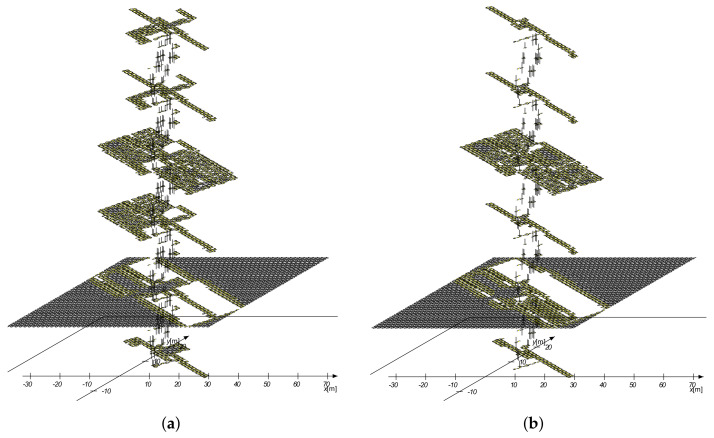
FootSLAM PM created from (**a**) the known building layout and (**b**) the building layout estimated with the IPS and MACS. White within coded hexagons represents high counters, yellow stands for medium counters, and black indicates low counters. Hexagons located on walls or not considered (closed) rooms had no prior (white hexagons).

The FootSLAM PMs created from the angular PDFs using an available floor plan and the 3D building reconstruction are given in Figure 8a,b, respectively. From this Figure, one can see six different floor levels, starting from the basement as the lowest level and reaching the fourth floor. The map contains the counters for each of the eight hexagonal prism edges coded using colors: white within coded hexagons represents high counters, yellow stands for medium counters, and black indicates low counters. Hexagons located on walls or not considered (closed) rooms had no prior (white hexagon). Since we used a rectangular hexagonal prism map, the ground floor also contains values outside the building, whereas there was naturally no prior outdoors for all the other levels. It should be noted that information about the outdoor environment like walkable paths could be added to the map before applying diffusion so that the map could also contain environmental information.

From the results, one can see that the original floor plan sometimes contained more information about the building layout: the balconies and the part of the floor plan on the first floor were additionally available. It should be noted that for simplicity, we only used the known floor plan, where we also performed experiments (the rooms on the first and second floor, the large room on the ground floor). Moreover, the 3D reconstruction algorithm only estimated the rooms of the second floor.

### 3.3. Error Rates for the Use of Different Map Combinations

**Figure 9:**
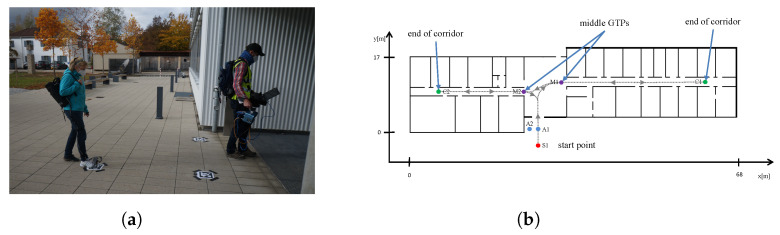
(**a**) Measurement equipment used within the experiment: six inertial sensors mounted on the foot (left) and hand-held IPS (right); (**b**) the position of the GTPs within the ground floor of our office building.

In our experiments, a pedestrian wearing six sensors on their foot (see Figure 9a, on the left) entered the building from outdoors. This figure also shows a second pedestrian on the right who scanned the interior of the building with a hand-held IPS. In this walk, we performed both scanning with the IPS and the measurement of inertial data. The pedestrian wearing the inertial sensors walked through the building (six different floor levels) and passed the ground-truth points (GTPs) located in the middle of the building and at the end of each corridor (see also Figure 9b, which shows the GTPs of the ground floor).

**Figure 10:**
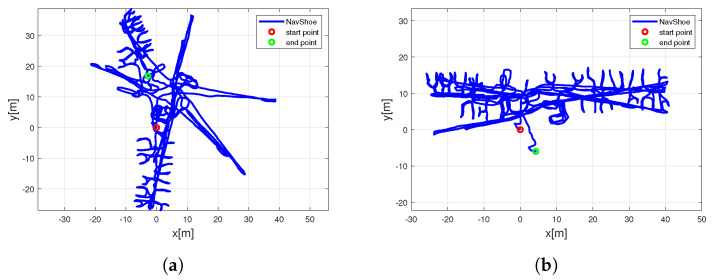
NavShoe x, y locations of the long walk for (**a**) Xsens MTw sensor 1 and (**b**) Xsens MTi600 sensor 6. One can see high sensor drift differences between both sensor types.

Two walks were performed with six sensors mounted on the foot, so that we measured a total of 12 different trajectories. Due to the fact that each sensor performed differently, the trajectories differed a lot even if the sensors were carried by the same person. Therefore, we expected a performance similar to if six different pedestrians walked the same trajectory. We passed all floors, starting with the ground floor, followed by the basement, and after that passing level 1, level 2, level 3, and level 4. The shorter walk intentionally stopped after the fourth floor to avoid walking the same paths back. In the long walk, the pedestrian continued walking downstairs and left the building until finally reaching the end point, which was the same as the starting point. The durations of the two walks were 25.8 and 54.2 min, and the estimated distances were 0.78km and 1.3km, respectively. In the second walk, the pedestrian entered additional rooms on levels 1 and 2. This took a while, because he also scanned the rooms during that walk. Therefore, he walked slowly and sometimes waited until the scanner finished the room. The GTPs passed were {S1, A1, 0M1, 0C1, 0M1, 0M2, 0C2, -1M1, 1C1, 1M1, 1C2, 2C2, 2M2, 2C1, 3C1, 3M1, 3C2, 4C2, 4M1, 4C1, 3M1} and {S1, A2, A1, 0M1, 0C1, 0M1, 0M2, 0C2, -1M1, 1C1, 1M1, 1C2, 2C2, 2M2, 2C1, 3C1, 3M1, 3C2, 4C2, 4M1, 4C1, 3M1, 2M1, 1M1, A1, A2, S1}, where the numbers preceding the GTP label indicate the floor level (−1 stands for basement, 0 for ground floor, 1 for first floor, etc.). The raw inertial sensor data were processed by the NavShoe algorithm (a lower UKF). Five of the six sensors were Xsens MTw sensors, and one of them was a Xsens MTi600 sensor, which offered a better accuracy—visible with a lower x, y, and z drift. This is shown in Figure 10a,b, where the x,y coordinates of the NavShoe results are shown for MTw sensor 1 and for MTi600 sensor 6, respectively. In the upper FootSLAM algorithm, we applied 10,000 particles and 0.5 m for the hexagon radius. We assumed that the starting conditions for position and heading were known and aligned the start of the NavShoe results to zero for both parameters.

The FootSLAM localization results were provided using the PM created from the building scan estimated with the IPS and MACS—for short, BSPM (building scan PM), based on the known floor plan (KFPM), and with no prior map (NoPM). The results in terms of error rates are given in Table 2 for the first walk. The errors were calculated using the Euclidean distance at the GTPs. From this table, one can see that the best mean error rate was 0.5 m, with a maximum error of 1.26 m if the KFPM was applied. It should be noted that FootSLAM sometimes resulted in higher errors at the end of the corridors, when there was drift and the algorithm searched for the best path until it reached convergence after turning back to the middle of the building. This caused higher “real-time” errors, especially at the end of the corridors, which were corrected afterwards when the map converged. For the BSPM, the mean error degraded slightly (mean 0.83 m, maximum 1.85 m), presenting very good values and showing that the use of the 3D reconstructed map was helpful. When we applied no PM, the quality degraded further to a mean value of 1.11 m and a maximum error of 2.94 m. The results were better for sensor 6, because it was of better quality in terms of drift. The z error was zero in all cases. We assumed that the floor separation was known and applied the so called Height Update (HUPT) [54] with stairs detection within the NavShoe based on height changes instead of pitch.

**Figure 11:**
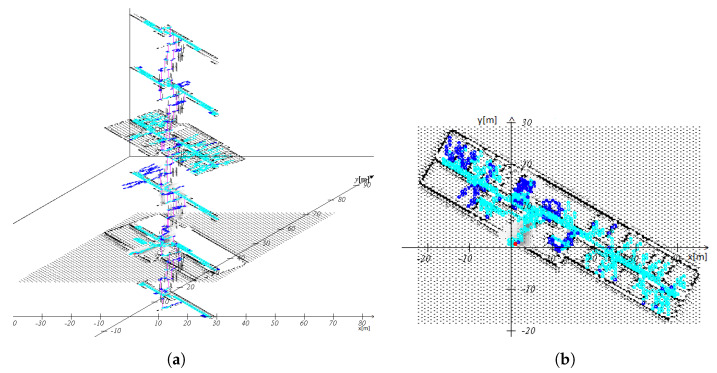
Resulting FootSLAM map of a walk in our office building using the prior map created from the building layout estimated with the IPS and MACS. The results show that the pedestrian walked through all six floor levels ((**a**) side view) and finally ended up outdoors on the ground-floor level (see red dot in (**a**,**b**), side and top view). On floor levels 1 and 2, he entered different rooms. Light blue indicates areas where the pedestrian passed hexagons provided by the prior map, and dark blue represents (new) explored areas.

The 3D FootSLAM results of the long walk (second walk) based on the BSPM are given in Figure 11. One can see that FootSLAM converged to the underlying building layout and finally returned to the outdoor starting point (red dot). From the 2D mapping of the results in Figure 11b, one can see that the estimated FootSLAM map precisely followed the floor and room structure of the building.

The error rates for the second walk are shown in Table 3. The mean results for the BSPM were similar to the results of the first walk, but the maximum error was slightly increased. The reason for this was that the SLAM searched for the best solution without having long loop closures on the second floor, where many rooms were entered. For the KFPM, we obtained similar results, except for sensor 2, which resulted in a higher mean error.

The main reason for this was again that FootSLAM searched for convergence at GTP 2C1, so that the real-time error was high at this point. Such an error increase can occasionally occur when using different kinds of PMs, and the walk was very difficult within the second half of the second floor. After reaching GTP 2M2 the first time, the algorithm searched for convergence, which may have led to very high errors, especially when using NoPM (see Table 3, column 4). Only after reaching convergence was this corrected. In order to show this, we also provide the non-real-time (non-RT) errors for NoPM in the table (all other values indicate real-time errors). The mean errors for non-RT NoPM were a little higher than 1m, which was similar to the results for the first walk. The maximum errors were higher than for the first walk due to convergence issues, as mentioned above. Finally and as expected, in all cases the errors were higher for the Xsens MTw sensors compared to the Xsens MTi600 sensor.

In our experiments, we realized that the erroneous wall in the middle of the building (refer to Section 3.1.2) in particular disturbed the results after the second floor. Moreover, we had to reduce the prior map weakening factor (PMWF), which made the results using the BSPM in particular more stable—but also a little less accurate. With a high PMWF, FootSLAM sometimes did not converge to the BSPM, and the map was disturbed after the middle of the second floor. This resulted in a high average error. Reducing the PMWF—i.e., applying the map less strongly—helped to overcome this problem. Finally, it should be noted that the doors were not provided on floor level 1 for the BSPM. This also caused uncertainties in the final decisions of the SLAM, but in all cases, the SLAM could handle it.

Within FootSLAM, we had to adapt the following parameters: the uncertainty of the scale $\sigma_{scale}$ to account for inaccuracies in the scale of the MTw sensor, the heading uncertainty $\sigma_{heading}$ to put more trust in the better sensor MTi600 in terms of heading, and the PMWF to account for different PM qualities. The parameters are given in Table 4. $\sigma_{scale}$ was enlarged for the MTw sensors (sensors 1–5), because they were not as accurate as the MTi600 sensor 6. When using the PM, it was better to add uncertainty to the scale for sensors 1–5, because otherwise the trajectory did not perfectly fit the underlying map. When using NoPM, it was better to set $\sigma_{scale}$ to zero, because it was already uncertain within the map finding process, i.e., convergence was reached faster without adding additional uncertainty. The additional uncertainty introduced by $\sigma_{scale}$ was reduced by the use of the underlying PM. $\sigma_{heading}$ was adjusted so that it accounted for the different heading drifts of the sensors. As discussed above, a smaller PMWF was used for the BSPM.

### 3.4. 3D Visualization

**Figure 12:**
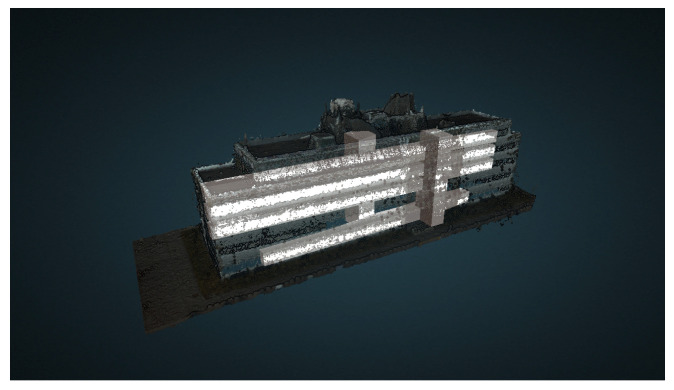
Combined view of 3D point cloud (from aerial images) and reconstructed building interior (from the IPS) of the scanned office building.

As described in Section 2.5, we displayed all data products together in a customized version of Potree-viewer. The outer hull was represented by more than 26.1 million reconstructed points together with the interior extracted from the indoor positioning system represented by 144 planes (see Figure 12).

We prepared the custom-built viewer to visualize the real-time positioning data of the FRs moving through the scene. This feature will be of great use for further projects and applications.

## 4. Discussion

First, we reconstructed our office building with visual sensors and converted the geo-referenced information into a PM suitable for FootSLAM. By visually comparing the results, we could observe that not all walls were reconstructed correctly, but the door openings were successfully determined. The differences were mainly due to walls made out of glass and a cupboard in the kitchen area. Second, we investigated the effect of using different kinds of PMs within the FootSLAM framework: (i) a PM resulting from 3D reconstruction; (ii) a PM resulting from a known floor plan; and (iii) no use of a PM, but the learning of a map of walkable areas simultaneously during the walk with the SLAM algorithm. According to consultations with FRs, this map built on hexagonal prisms was not very easy to interpret, and they mentioned that a visualization including 3D reconstruction results would be better. This was one of the reasons for combining the two tools: a 3D reconstruction for visualizing and estimating a PM, and a real-time SLAM based on light-weight sensors for localizing the FRs. The results showed that even an unreliable PM obtained by 3D reconstruction enhanced the accuracy of the FR localization algorithm. The results for the use of a known map were even better, and we could apply the map with a higher PMWF. The parameters had to be tuned for sensors of different qualities, which was manageable because the sensor type was known in advance, and the weight of the PM had to be adjusted, because errors within the map could occasionally cause non-convergence. Depending on the PM, this value was also known in advance. Therefore, these adjustments could easily be performed. It was also shown that the real-time performance with no available PM degraded. However, if we look at the error values from the final converged map, i.e., the non-RT NoPM error values, we can see that the errors were enhanced during the whole estimation process. In all cases, the map converged, so that the results were more and more refined when an area was explored twice—including same areas of different floors. To examine the algorithm convergence, we tested FootSLAM with different seeds for the long walk, sensor 1 and sensor 6, respectively, in order to test different sensor types. We performed 10 runs with different seeds and applied BSPM, KFPM, and NoPM. In all cases except for the combination of the BSPM and sensor 1, FootSLAM converged finally to the map. For the combination of sensor 6 and the BSPM, we also achieved convergence on all 10 runs. For the combination of sensor 1, the BSPM, and a PMWF of 0.01, FootSLAM did not converge three out of ten runs. We reduced the PMWF to 0.001, but FootSLAM still did not converge one out of ten runs. Therefore, we tested FootSLAM using the resulting map of sensor 6 learned from the BSPM to refine the map with walkable areas. This resulted in no convergence issues on all test runs for sensor 1. The combination of the BSPM—which could contain errors—with the map of walkable areas obtained solely by an inertial sensor—with which we were able to correct the errors of the BSPM—was in any case advantageous in terms of convergence. In our walks, we decided to visit each floor only once, and therefore we did not revisit areas, except between the two staircases and when walking back to the staircases. Another solution to overcome the problem of non-convergence, i.e., resulting in a map, e.g., outside the building, would be to add a “not allowed mark” to those areas that cannot be entered.

While being very accurate in terms of position error, the FootSLAM algorithm revealed the following limitations: The starting conditions were assumed to be known. The use of GNSS data to initialize and, e.g., the use of the visualization for initializing if no GNSS data were available have not yet been fully investigated and integrated. The system has to be tested in large halls to see if it also converges without any restrictions inside a room—if there are no obstacles to reduce walkable areas. The system could handle magnetometer data in the so-called MagSLAM extension in playback mode, but this was not tested in real time and it is computationally complex. While we tested FootSLAM for running and crawling, it lacks full testing and adaptation to different motion modes like climbing, crawling, and kicking.

Compared to the error figures of commercial state of the art systems, the achieved error rates were similar or even better. For instance, compared to the indoor.rs and oriient.me systems [26,29], our system achieved a similar performance: both claim that they have an accuracy within 1 m, which was also the case on average with our system, even without any prior knowledge of the building layout. A comparison to similar techniques designed for FRs that do not rely on prior knowledge of the building or on infrastructure is rather difficult. For example, in [55], the PERSY system achieved a 75% circular error probability (CEP) of a horizontal position error (HPE) of 8 m and a 75% linear error probability (LEP) of a vertical position error (VPE) of 3 m, but they added a no-broadcast error of 50 m and 15 m for HPE and VPE, respectively, and we do not know how often this value was finally added. In addition, these works tested other motion modes like crawling, which we did not test in this paper. Moreover, 16 indoor points and 3 outdoor points were passed using a GNSS receiver in their system. Even if it seems that we achieved better accuracy indoors, the comparison would not be fair for either the horizontal or vertical error, because we assumed that the starting conditions and height of the floor levels of the building were known. A comparison to Pointer’s method [32] with its 2 m accuracy reveals that we achieved a better performance with the advantage of a wider coverage.

Overall, we could show that the final localization and visualization were enhanced when we were able to use the reconstructed map within the processing tool chain.

## 5. Conclusions

In this paper, we combined 3D building reconstruction with 3D pedestrian localization and 3D visualization in a system designed for professional use cases like FRs in a police or fire brigade operation. The following properties are very important for these end-users: the visualization tool should be able to switch between levels, show walls instead of point clouds, and visualize the positions of several FRs, provided by a low-complexity and light-weight localization solution ensuring very good and stable localization results.

To provide real-time FR locations, we proposed the use of a technique solely based on a small and light-weight inertial unit worn on the shoe or at other locations on the body. The algorithm to reduce the remaining inherent drift applied in this paper was the FootSLAM algorithm. This algorithm could be enhanced with prior map information. We investigated the use of a prior map based on 3D reconstruction estimated by the MACS and IPS and compared it to the use of other prior information like the original map and/or the FootSLAM-estimated maps. The advantage of using a prior map provided by 3D reconstruction—assuming there is no knowledge of the building layout—is that it is geo-referenced and that the final localization and visualization are more valuable.

We presented the whole process for using 3D reconstruction within a self-localization technique and showed that we could achieve reliable self-localization with an average accuracy of below 1 m for our experiments with the use of prior maps and of 1–2 m without any prior map (non-real-time). It was also shown that the results for the use of a prior map created by 3D reconstruction were only slightly worse than when using the original (known) map. It is evident that a 3D reconstruction database would help to obtain more reliable self-localization data for first responders, especially in indoor scenarios, and we provided the whole processing chain for using this system in an appropriate way, i.e., in a probabilistic sense, within this paper. Future research will use the point clouds of the 3D reconstruction directly for angular PDF calculations in order to avoid hard decisions on walls. In addition, we will further investigate the use of magnetic data in real time, GNSS data for seamless outdoor/indoor navigation and initialization, and different motion modes. The entire system, which has already been demonstrated to and tested with end-users, will be tested further at different locations.

## Figures and Tables

**Table 1:** Euclidean distance between 3D reconstruction and known floor plan for the four corners A, B, C, and D of the building in Figure 7a.

$d_{A}$ (m)	$d_{B}$ (m)	$d_{C}$ (m)	$d_{D}$ (m)
0.27	0.22	0.26	0.12

**Table 2:** Mean error (mean) and maximum error (max) of the first walk with six different sensors mounted on the shoe. The pedestrian stopped at different GTPs, and the error was measured at the GTPs. The results are shown for different PMs: BSPM, KFPM, and NoPM in real time (RT). The units are meters.

	BSPM		KFPM		NoPM	
Sensor	Mean (m)	Max (m)	Mean (m)	Max (m)	Mean (m)	Max (m)
1	0.76	1.63	0.47	1.06	1.08	3.43
2	0.9	1.7	0.49	1.24	1.16	3.87
3	0.8	1.5	0.49	1.19	1.22	2.91
4	0.94	1.88	0.53	1.18	1.17	2.52
5	0.85	1.51	0.58	1.38	1.08	2.73
6	0.74	2.9	0.44	1.53	0.94	2.17
mean	0.83	1.85	0.5	1.26	1.11	2.94

**Table 3:** Mean error (mean) and maximum error (max) of the second walk with six different sensors mounted on the shoe. The pedestrian stopped at different GTPs, and the error was measured at the GTPs. The results are shown for different PMs: real-time (RT) BSPM, KFPM, NoPM, and non-RT NoPM.

	BSPM		KFPM		NoPM		Non-RT NoPM	
Sensor	Mean	Max	Mean	Max	Mean	Max	Mean	Max
	(m)	(m)	(m)	(m)	(m)	(m)	(m)	(m)
1	0.78	1.69	0.62	2.27	2.4	23.42	1.45	2.58
2	1.01	3.49	1.53	3.81	2.0	14.68	1.27	2.58
3	0.84	2.12	0.61	2.24	1.81	21.59	0.91	2.82
4	0.74	1.58	0.6	2.04	2.66	22.21	1.76	3.8
5	0.79	1.7	0.62	2.46	2.0	21.22	0.85	2.25
6	0.61	1.61	0.58	1.56	1.71	19.12	0.74	1.59
mean	0.8	2.03	0.76	2.4	2.1	10.37	1.16	2.6

**Table 4:** Parameters for FootSLAM depending on the sensor and PM: heading uncertainty $\sigma_{heading}$, uncertainty of the scale $\sigma_{scale}$, and PM weakening factor (PMWF).

	$\sigma_{\mathrm{scale}}$ (m)	$\sigma_{\mathrm{heading}}$ (rad)	PMWF
BSPM, MTw	0.05	0.6	0.01
BSPM, MTi600	0.0	0.3	0.01
KFPM, MTw	0.05	0.6	0.1
KFPM, MTi600	0.0	0.3	0.1
NoPM, MTw	0.0	0.6	N.A.
NoPM, MTi600	0.0	0.3	N.A.

## Data Availability

The data presented in this study are available on request from the corresponding author. The data are not publicly available due to privacy issues.
